# Rotavirus VP6 as an Adjuvant for Bivalent Norovirus Vaccine Produced in *Nicotiana benthamiana*

**DOI:** 10.3390/pharmaceutics11050229

**Published:** 2019-05-11

**Authors:** Maria Malm, André Diessner, Kirsi Tamminen, Markus Liebscher, Timo Vesikari, Vesna Blazevic

**Affiliations:** 1 Vaccine Research Center, Faculty of Medicine and Health Technology, Tampere University, Biokatu 10, FI-33520 Tampere, Finland; maria.malm@tuni.fi (M.M.); kirsi.tamminen@tuni.fi (K.T.); timo.vesikari@tuni.fi (T.V.); 2 Icon Genetics GmbH, Weinbergweg 22, 06120 Halle, Germany; Diessner@icongenetics.de (A.D.); Liebscher@icongenetics.de (M.L.)

**Keywords:** rotavirus, VP6, adjuvant, bivalent vaccine, norovirus, VLP, plant-production, blocking antibodies

## Abstract

Rotaviruses (RVs) and noroviruses (NoVs) are major causes of childhood acute gastroenteritis. During development of a combination vaccine based on NoV virus-like particles (VLP) and RV VP6 produced in baculovirus expression system in insect cells, a dual role of VP6 as a vaccine antigen and an adjuvant for NoV-specific immune responses was discovered. Here the VP6 adjuvant effect on bivalent GI.4 and GII.4-2006a NoV VLPs produced in *Nicotiana benthamiana* was investigated. BALB/c mice were immunized intradermally with suboptimal (0.3 µg) dose of each NoV VLP alone or combined with 10 µg of VP6, or equal doses of NoV VLPs and VP6 (1 µg/antigen). NoV-specific serum IgG antibodies and their blocking activity were analyzed using vaccine-homologous and heterologous NoV VLPs. Immunization with 0.3 µg NoV VLPs alone was insufficient to induce NoV-specific immune responses, but with co-administration of 10 µg of VP6, antibodies against vaccine-derived and heterologous NoV genotypes were generated. Furthermore, corresponding adjuvant effect of VP6 was observed with 1 µg dose. Efficient uptake and presentation of VP6 by dendritic cells was demonstrated in vitro. These results show that adjuvant effect of VP6 on bivalent NoV VLP vaccine is independent of the cell source used for vaccine production.

## 1. Introduction

Norovirus (NoV) infections are the most common cause of acute gastroenteritis worldwide across all age groups, causing estimated 685 million cases of acute gastroenteritis (AGE) each year and being responsible for 50,000 yearly deaths of children under five years of age [1,2]. There is no vaccine available for NoV, but promising candidates are being developed and tested in clinical and preclinical phases [3,4]. However, in countries where rotavirus (RV) vaccination has not been introduced, RV infections are still the most common cause of AGE-related morbidity and mortality of young children [5]. Oral RV vaccines have demonstrated good efficacy in high income countries, while in low-income settings, where RV disease is the most severe, the effectiveness of vaccines is lower [6].

NoV major capsid protein, VP1, spontaneously self-assembles to form VLPs that are successfully used as virus particle surrogates for vaccine development [3,7,8,9]. Until recently, there has been no appropriate method for NoV propagation and despite recent advances e.g., with enteroid NoV culture system [10], manufacturing scale cultivation is still lacking. To date, there are at least 30 human NoV genotypes identified, based upon viral capsid (VP1) and RNA-dependent, RNA polymerase protein sequences. Most NoVs infecting humans belong to genogroup I (GI, 9 genotypes) and genogroup II (GII, 19 genotypes) [3], and the lack of cross-reactivity between GI and GII NoVs [8,11,12] suggests that inclusion of at least one VLP from GI and one from GII is necessary for cross-protective NoV vaccine. Indeed, the most advanced NoV vaccine in clinical trials [9] is a bivalent vaccine consisting of NoV GI.1 and GII.4 VLPs and aluminum hydroxide [Al(OH)_3_] as an adjuvant.

We have developed a trivalent combination vaccine candidate children as a primary target group against NoV and RV AGE, consisting of two NoV virus-like particles (VLPs), GII.4-1999 and GI.3 and oligomeric RV VP6 [7,8]. RV VP6, ~45 kDa in size, is the most abundant and highly immunogenic protein that forms the intermediate layer of the virion. Furthermore, RV VP6 displays a high degree of conservation among group A RVs that cause >90% of human RV infections [13]. VP6 forms different oligomeric nanostructures such as nanotubes and nanospheres in vitro, depending on conditions such as pH and ionic strength [14]. VP6 induced immune response has been shown to protect from homologous and heterologous RV infection in animal models [15,16,17,18]. Furthermore, positive correlation of serum IgA targeted to RV VP6 following both RV infection and vaccination has been observed in humans [19,20,21]. These characteristics make VP6 an ideal non-live RV vaccine candidate, but additionally, it has been demonstrated that RV VP6 has also an adjuvant effect on co-delivered antigens such as NoV VLPs [7,22,23,24,25,26]. We have previously shown that only when VP6 was co-administrated intramuscularly (IM) with suboptimal dose of either GI.3 or GII.4 NoV VLPs in mice, NoV-specific response was elicited [22,23]. This finding is of a high importance as an adjuvant-free vaccine is preferred for pediatric population.

In the present study, we evaluated RV VP6 adjuvant effect on a bivalent NoV GI.4 and GII.4-2006a VLP vaccine rather than monovalent VLPs studied before [22,23]. Also, in contrast to our previous studies using baculovirus (BV)-insect cell produced NoV VLPs and RV VP6 all vaccine antigens used here were produced using plant *Nicotiana* (*N.*) *benthamiana* expression system. Furthermore, in vitro assays using mouse primary bone-marrow-derived dendritic cells (BMDCs) were performed to test plant-derived VP6 interaction with antigen-presenting cells (APC).

## 2. Materials and Methods

### 2.1. Recombinant Proteins

Plant-derived GI.4 and GII.4-2006a NoV VLPs and RV VP6 were expressed in *N. benthamiana* plants using magnICON^®^ vector based on a tobacco mosaic virus (TMV) RNA replicon system, purified and characterized by ICON Genetics GmbH (Halle, Germany) [27,28,29,30]. Briefly, *N. benthamiana* plants were vacuum-infiltrated (80–100 mbar for 3–4 min) with diluted *Agrobacterium tumefaciens* cultures with TMV-based assembled magnICON^®^ vectors carrying codon-optimized VP1 (GI.4 or GII.4-2006a) or VP6 DNA cloned for expression and plant material was harvested 6–14 days post infiltration. Biomass was homogenized and clarified by centrifugation (20 min 15,000× *g*) and filtration (Millipore^®^ glass fiber filter AP25). Norovirus VLP were sedimented and purified by PEG precipitation and filtration. Rotavirus VP6 high molecular weight structures were purified using a combination of cation- and anion-exchange chromatography. Purified material was formulated in phosphate-buffered saline (PBS) (10 mM NaH_2_PO_4_, 137 mM NaCl), pH 7.3. VLP formation was confirmed by size exclusion-high-performance liquid chromatography (SE-HPLC) with light scattering analysis. SE-HPLC analysis was performed on an Agilent 1200 HPLC system (Agilent Technologies, Waldbronn, Germany) coupled to a multiangle light scattering detector (MALS-detector) with a quasi-elastic light scattering dynamic light scattering module (QELS-DLS module) and a refractometer (all Wyatt Technologies Europe, Dernbach, Germany). Protein concentration was measured by bicinchoninic acid assay and protein purity was determined with reduced capillary gel electrophoresis performed on an Agilent 2100 bioanalyzer using a 230 Protein Kit and 2100 Expert Software (Agilent Technologies, Waldbronn, Germany). Endotoxin level was quantitated by endpoint chromogenic Limulus Amebocyte Lysate (LAL) test (QCL-1000, Lonza, Walkersville, MD, USA) and residual host cell DNA contamination by Quant-iT dsDNA High Sensitivity Kit (Thermo Fisher Scientific, Waltham, MA, USA). High-order structures and morphology of the proteins were imaged by Zeiss EM900 Transmission Electron Microscope (TEM) (Carl Zeiss Microscopy, Jena, Germany). Samples were collected on cooper grids and contrasted with 2% uranyl acetate. Micrographs were taken with a Variospeed slow scan camera (SM-1k-120, Tröndle, Germany) using the iTEM software from Olympus SIS (Münster, Germany). Proteins were stored at +4 °C until use and diluted in PBS pH 7.3 (Lonza BioWhittaker, Walkersville, MD, USA, Cat. BE17-516F) and mixed at desirable concentrations for immunizations. Mock preparation of *N. benthamiana* (magnICON^®^) served as a control antigen for immunological assays. Other NoV protein antigens used for analytical methods; GI.1, GI.3, GII.4-1999, GII.4-2010 NO, GII.4-2012 Sydney, and GII.12 VLPs, were produced in Sf9-cells utilizing baculovirus-insect cell expression system, purified and characterized as described earlier by our group [7,31,32].

### 2.2. Immunization of Mice

The groups of five female BALB/c OlaHsd mice (Envigo, Horst, The Netherlands) 7 weeks of age, were used for experimental and control immunizations according to Table 1. Vaccine formulations were administrated intradermally (ID) at the base of the tail, dorsal side of the mouse. Mice were immunized twice at day 0 and day 21 and terminated at day 35, according to our standard procedure [7,8,22]. Two different doses (0.3 µg or 1 µg) of a bivalent combination of GI.4 and GII.4-2006a NoV VLPs were tested alone (Gr I and III, Table 1), or mixed with RV VP6. The adjuvant effect of RV VP6 was first evaluated by combining 10 µg VP6 with suboptimal doses of NoV VLPs (0.3 µg, Gr II) and then by administrating equal amounts (1 µg) of each antigen as a mixture (Gr IV). Control mice (Gr V) received 50 µL of phosphate-buffered saline (PBS) carrier only.

Animals were anesthetized with sevoflurane (Baxter Healthcare Ltd., Deerfield, IL, USA, Cat. FDG9117) inhalation prior to immunization and intraperitoneally with a formulation of medetomidine (Dorbene^®^ vet, Laboratorios SYVA S.A., Leon, Spain, Cat. 067632) and ketamine (Ketaminol^®^ vet, Intervet International B.V., Boxmeer, The Netherland, Cat. 511485) for euthanasia. Blood samples were collected at the time of termination [33]. All of the experimental procedures carried out were in accordance with the regulations and guidelines of the Finnish National Experiment Board (permission number ESAVI/10800/04.10.07/2016) and mice welfare was monitored throughout the experiment on a daily basis.

### 2.3. Serum IgG, IgG1, and IgG2a ELISA

Vaccine-induced NoV GI.4-, GII.4-2006a-, and RV VP6-specific antibodies were determined in termination blood serum samples according to the previously published procedures by enzyme-linked immunosorbent assay (ELISA) [7,8]. Type-specific antibodies were analyzed on Corning high binding 96-well half-area microplates (Corning Inc., Corning, NY, USA, Cat. 3690) coated with 2 µg/mL of the plant-produced protein antigens. Two-fold diluted individual mouse sera starting at 1:200 dilution were plated on blocked (5% milk in PBS) plates and bound antibodies were detected with horseradish peroxidase (HRP)-conjugated goat anti-mouse IgG (Sigma-Aldrich, Saint Louis, MO, USA, Cat. A4416), IgG1 (Invitrogen, Carlsbad, CA, USA, Cat. A10551) or IgG2a (Invitrogen, Cat. A10685), followed by a reaction with SIGMAFAST o-phenylenediamine dihydrochloride (OPD) substrate (Sigma-Aldrich, Saint Louis, MO, USA, Cat. P9187). Optical density at 490 nm (OD490) was measured with Victor2 1420 microplate reader (Wallac, Perkin Elmer, Waltham, MA, USA). The wells of control mouse sera were used to determine the cut-off value (mean OD490 + three standard deviations (SD)). Specimens with a net OD490 above the set cut-off value and >0.1 were considered positive. The end-point antibody titers of sera were defined as the highest sample dilution with an OD490 above the set cut-off value. The serum IgG mock responses to *N. benthamiana* were tested accordingly on plates coated with 2 µg/mL with mock preparation *N. benthamiana* (magnICON^®^). To measure cross-reactive NoV-specific IgG antibodies in the termination sera, group-wise pooled sera were 1:200 diluted and analyzed on plates coated with 1.0 µg/mL of baculovirus-produced GI.1, GI.3, GII.4-1999, GII.4-2010 New Orleans (NO), GII.4-2012 Sydney (SYD), or GII.12 VLPs following the procedure described above.

### 2.4. Blocking Assays

The presence of serum IgG antibodies that block binding of NoV VLPs to the HBGA carbohydrates were determined in ELISA-based blocking assay according to previously published protocols [23]. Group-wise pooled mouse sera were examined for capability to block VLP binding on HBGAs present in pig gastric mucin (PGM, type III, Sigma-Aldrich, Saint Louis, MO, USA, Cat. M1778) [23,34]. Serum two-fold dilutions were pre-incubated with 0.1 µg/mL GI.4 or GII.4-2006a NoV VLPs in sample buffer (1% milk in PBS + 0.05% tween) prior to plating on PGM-coated (2 µg/mL) and blocked (5% milk in PBS) microwell plates (Corning Inc., Corning, NY, USA, Cat. 3690). Following 1 h incubation at +37 °C, bound VLPs were detected with rabbit polyclonal anti-NoV antisera (ICON Genetics, Halle, Germany) followed by anti-rabbit IgG-HRP antibody (Abcam, Cambridge, UK, Cat. ab97051) and OPD substrate. Maximum HBGA binding of VLPs was determined in wells with VLPs lacking the serum. The blocking index (%) was calculated as follows: 100% − [(OD490 of wells with VLP and serum/OD490 of maximum binding wells) × 100%]. A 50% blocking titer (BT_50_) was determined as the reciprocal of the highest serum dilution able to block at least 50% of VLP-HBGA binding.

### 2.5. VP6 Internalization and Intracellular Staining

Uptake of plant-based RV VP6 nanotubes by BMDCs was analyzed by intracellular staining and flow cytometry following the method previously described for immortalized mouse cell lines [24] with slight modifications. BMDCs were plated to non-treated multidish wells in CM containing 100 µg/mL of VP6 nanotubes or CM alone (for untreated control cells) and incubated at 37 °C, 5% CO_2_ for 20 h. After the incubation the cells were harvested and culture supernatants collected and stored at −80 °C for cytokine analysis. Cells were washed (PBS + 3% FBS) and blocked for non-antigen-specific binding of immunoglobulins to the FcγIII and FcγII with rat anti-mouse CD16/CD32 Fc Block (Clone 2.4G2, Becton Dickinson, Franklin Lakes, NJ, USA, Cat. 553142). Cells were treated with BD Cytofix/Cytoperm Plus kit (Becton Dickinson, Cat. 555028) according to manufacturer’s instructions prior to staining RV VP6 intracellularly with rabbit polyclonal rotavirus group A antibody (Genway Biotech Inc., San Diego, CA, USA, Cat. GWB-459FC9) followed by fluorescein isothiocyanate (FITC)-conjugated goat anti-rabbit Ig (Becton Dickinson, Franklin Lakes, NJ, USA, Cat. 554020). Cells were resuspended in FACS Staining Buffer (Becton Dickinson, Franklin Lakes, NJ, USA, Cat. 554657) for acquisition and analysis using FACS CantoII flow cytometer and FACSDiva Software V 6.1.3. The data analysis was performed using FlowJo software version 10.1.

### 2.6. Cytokine Analysis

Quantities of tumor necrosis factor alpha (TNF-α) and interleukin-6 (IL-6) cytokines in the BMDC culture supernatants were determined by commercial ELISA kits, Mouse TNF-α DuoSet (R&D Systems, Minneapolis, MN, USA, Cat. DY410-05) and Mouse IL-6 DuoSet (Cat. DY406-05) according to the manufacturers’ instructions as previously described [24]. Victor^2^ 1420 Multilabel Counter (Wallac, PerkinElmer, Waltham, MA, USA) plate reader was used for optical density reading (OD) of the plate. For each assay the background signal from blank wells (wells without supernatant) was subtracted from all of the OD readings on the plate. Standard curves were plotted and used for calculating the cytokine concentration of each sample (pg/mL).

### 2.7. ELISPOT IFN-γ Assay

To confirm VP6 antigen uptake and processing by antigen-presenting cells (APC), an ELISPOT IFN-γ assay was applied. First, BALB/c mouse bone marrow-derived DCs (BMDC) were generated and pulsed with purified, plant-derived VP6 protein according to previously published method [35]. For pulsing, thawed and washed BMDCs were plated at 2 × 10^6^ cells/mL on non-treated cell-culture 24-well plates (Corning Costar, Corning, NY, USA, Cat. CLS3738) in cell medium (CM, RPMI-1640, Cat. R0883, supplemented with 100 U/mL penicillin and 100 µg/mL streptomycin, Cat. P0781, 2 mm L-glutamine, Cat. G7513 and 10% fetal bovine serum (FBS), Cat. F9665, all from Sigma-Aldrich and 50 µm 2-mercaptoethanol, Gibco 31350-010). BMDCs were incubated ~22 h at +37 °C and 5% CO_2_ with 100 µg/mL VP6, and control BMDCs (unpulsed) were incubated in CM only.

RV VP6-specific T cell responses were analyzed using previously published enzyme-linked immunospot (ELISPOT) IFN-γ assay [8,35]. 96-well MultiScreen HTS-IP filter plates (Millipore, Billerica, MA, USA, Cat. MSIPN 4W50) were coated with anti-mouse IFN-γ (Mabtech Ab, Nacka Strand, Sweden, Cat. 3321-3), washed and blocked with 10% FBS in CM before plating the antigens and cells. Splenocytes (0.2 × 10^6^ cells/well) of mice immunized with 10 µg BV-derived VP6 in a previous study were used as responder cells in the assay. The responder cells were mixed with VP6-pulsed or control BMDCs at three different ratios (40:1, 20:1 or 10:1) Concanavalin A (Con A; Sigma-Aldrich, Saint Louis, MO, USA) 10 µg/mL was used as a positive control to stimulate IFN-γ secretion from splenocytes. Plates were incubated for ~22 h at +37 °C and 5% CO_2_, and the spots were developed with biotinylated anti-mouse IFN-γ monoclonal antibody (Cat. 3321-6), 0.5 µg/mL in PBS/0.5% FBS, 2 h at RT, and alkaline-phosphatase (ALP) conjugated streptavidin (Cat. 3310-10) reacting with BCIP/NBT substrate (Cat. 3650-10, all from Mabtech, Nacka Strand, Sweden). The spots were counted by an ImmunoSpot^®^ automatic CTL analyzer (CTL-Europe GmbH) and the results are expressed as mean spot forming cells (SFCs) per 10^6^ live splenocytes of replicate wells. For each IFN-γ response to the pulsed BMDC the positivity cut-off was calculated as a mean SFC/10^6^ cells of wells with control BMDC + 3 × SD.

### 2.8. Statistics

A nonparametric Mann-Whitney U-test was employed to assess the statistical differences between observations of two independent groups. Data was analyzed with GraphPad Prism version 8.0.1. The statistically significant difference was defined as *p* < 0.05.

## 3. Results

### 3.1. Protein Expression and Morphology

**Figure 1:**
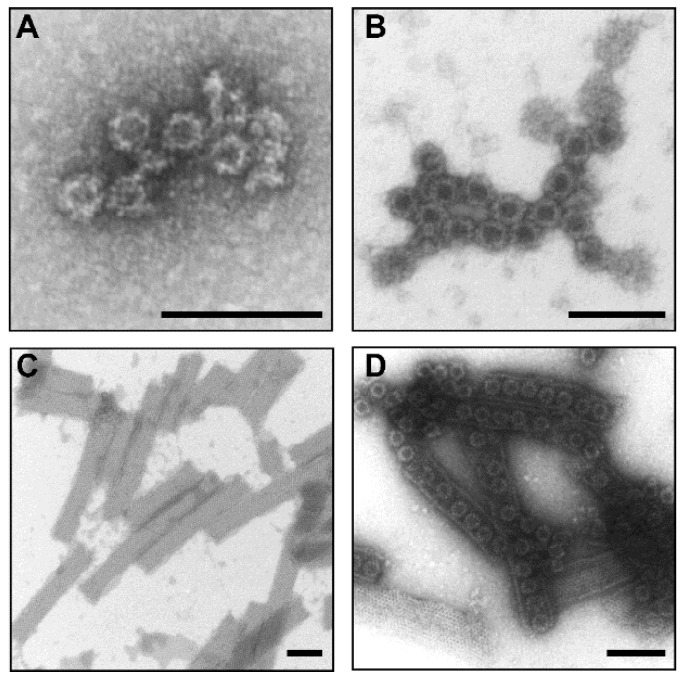
Electron micrographs of the highly purified plant-produced norovirus (NoV) (**A**) GI.4 VLPs, (**B**) GII.4-2006a VLPs, (**C**) recombinant RV VP6 protein and (**D**) a mixture of the aforementioned examined by transmission electron microscope EM900 (Carl Zeiss Microscopy, Germany) following negative staining with 2% uranyl acetate. The black bar corresponds to 100 nm.

Plant-produced NoV GI.4 and GII.4-2006a VLPs and RV VP6 protein purity were verified as described in the Material and Methods (data not shown). The integrity and morphology of protein nanoparticles were confirmed under TEM (Figure 1A–C). NoV GI.4 (Figure 1A) and GII.4-2006a (Figure 1B) VP1 capsid proteins had assembled into VLPs and most of the VP6 trimers were associated into nanotubes (Figure 1C,D).

### 3.2. Immune Responses Induced with Suboptimal Doses of Bivalent Nov VLP Alone or Combined with RV VP6 in Excess

#### 3.2.1. Serum NoV Genotype-Specific IgG, IgG1 and IgG2a

**Figure 2:**
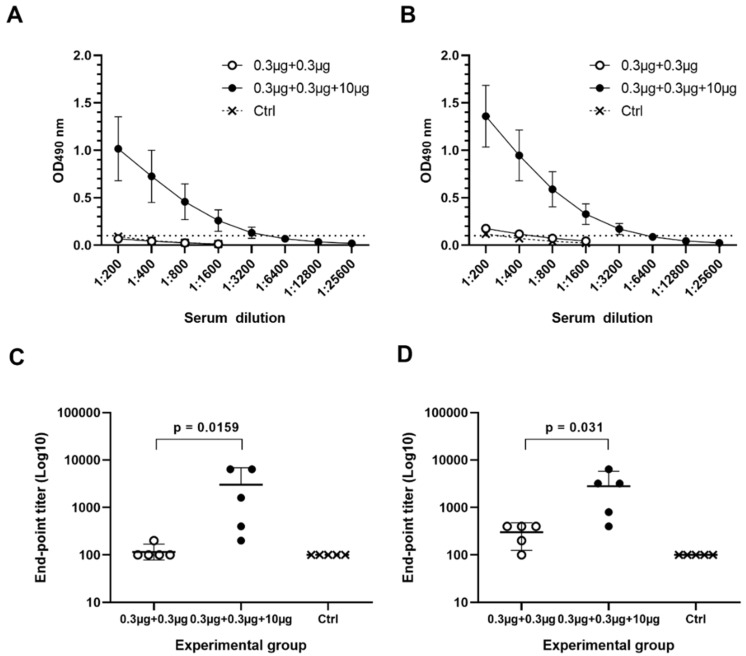
Serum norovirus (NoV)-specific IgG titration. Individual termination serum of each mouse in experimental groups immunized with 0.3 µg NoV VLPs alone or mixed with 10 µg of VP6, and the control group (Ctrl, PBS only) were two-fold diluted for analyzing NoV (**A**) GI.4- and (**B**) GII.4-2006a-specific IgG antibodies. Shown are mean titration curves for each group and standard errors of the mean. Dashed line indicates positivity cut-off (OD > 0.1). IgG end-point titers against (**C**) GI.4 and (**D**) GII.4-2006a VLPs were determined as the reciprocal of the highest serum sample dilution giving a positive reading. Shown are geometric mean titers with 95% confidence interval of each group. Groups were compared by Mann–Whitney U-test and *p* values were determined.

Immunization of mice using suboptimal dose of 0.3 µg NoV GI.4 and GII.4-2006a VLPs as a mixture (Gr I, Table 1) did not induce serum IgG to GI.4 (Figure 2A) nor to GII.4-2006a (Figure 2B). Adding 10 µg of RV rVP6 protein into the mixture (Gr II, Table 1) remarkably improved NoV-specific IgG responses to both GI.4 (Figure 2A) and GII.4-2006a (Figure 2B), with significantly increased geometric mean titers (GMTs), 1393 for GI.4 (Figure 2C) and 1838 for GII.4-2006a (Figure 2D). NoV-specific IgG was not detected in any of the control animal sera (Figure 2A–D).

**Figure 3:**
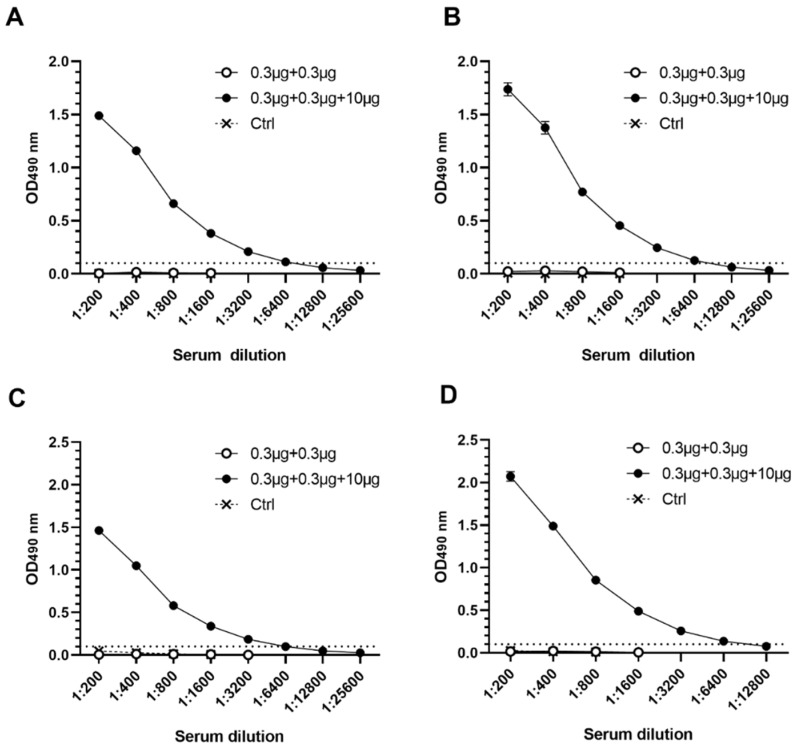
Serum norovirus (NoV)-specific IgG1 and IgG2a subtypes. Pooled termination sera of experimental groups immunized with 0.3 µg NoV GI.4 and GII.4-2006a VLPs alone or mixed with 10 µg VP6, and the control group (Ctrl, PBS only) were two-fold diluted for analyzing NoV (**A**,**C**) GI.4 and (**B**,**D**) GII.4-2006a-specific IgG1 and IgG2a. Shown are titration curves with standard error of the mean. Dashed line indicates positivity cut-off (OD ≥ 0.1).

Analysis of GI.4- and GII.4-2006a -specific serum IgG1 (Figure 3A,B) and IgG2a (Figure 3C,D) showed that addition of VP6 in the mixture induced increase in both IgG1 and IgG2a subtypes, resulting in well-balanced Th1/Th2 profile against both NoV genotypes.

To confirm the success of the immunization, all mice receiving the VP6 developed strong IgG antibody response to the protein (data not shown). No positive IgG antibody responses against mock preparation *N. benthamiana* (magnICON^®^) were observed in any of the mice immunized with the plant-produced proteins (data not shown).

#### 3.2.2. Serum NoV-Specific Cross-Reactive IgG Antibodies

**Figure 4:**
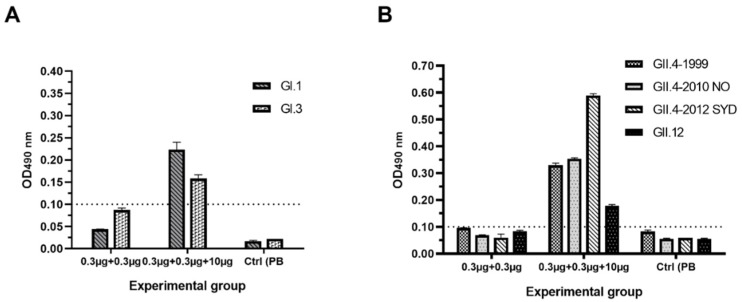
Serum norovirus (NoV)-specific cross-reactive IgG antibodies. Pooled termination sera of experimental groups receiving 0.3 µg NoV GI.4 and GII.4-2006a VLPs alone or mixed with 10 µg of RV VP6 were diluted 1:200 and tested for cross-reactive IgG against two NoV (**A**) GI VLPs and (**B**) four GII VLPs. Control (Ctrl) mice received carrier only (PBS). Mean OD values with the standard errors are shown for replicate analysis.

The presence of cross-reactive IgG antibodies in pooled sera of immunized experimental and control mice was measured against two VLPs representing NoV GI (Figure 4A) and four VLPs representing GII (Figure 4B). Following administration of bivalent suboptimal dose (0.3 µg per Ag) of NoV VLPs alone, cross-reactive IgG antibodies remained under detection limit (OD < 0.1). When combining 10 µg VP6 with NoV VLPs, cross-reactive IgG level against all six tested VLPs genotypes could readily be detected.

#### 3.2.3. Antibodies Block NoV VLP Binding to PGM HBGAs

**Figure 5:**
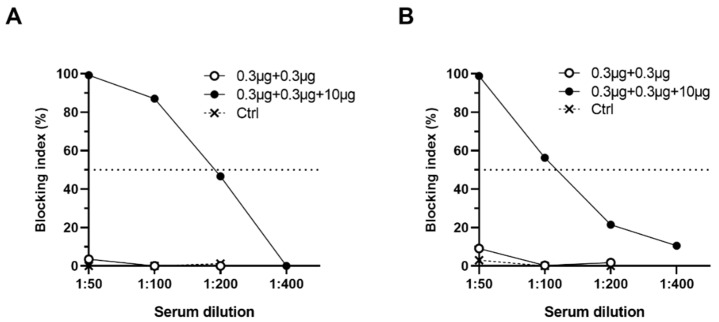
Blocking of norovirus (NoV) VLP binding to histo-blood group antigens (HBGAs). Two-fold diluted, group-wise pooled sera of mice immunized with 0.3 µg NoV GI.4 and GII.4-2006a VLPs alone, or mixed with 10 µg VP6, and the control (Ctrl) mice sera were tested for potential to block binding of NoV (**A**) GI.4 or (**B**) GII.4-2006a VLPs to HBGAs present in pig gastric mucin (PGM). Shown are group mean blocking index (%) with standard error of the mean. Horizontal dashed line represents 50% blocking.

Serum NoV-specific antibodies able to block VLP-HBGA binding were determined of mice sera using PGM HBGA-based blocking assay (Figure 5A,B). None of the mice immunized with suboptimal VLP dose alone or control mice had blocking antibodies at serum dilution 1:50. The administration of suboptimal dose of NoV VLPs combined with 10 µg RV VP6 generated homologous PGM blocking at level against GI.4 (Figure 5A) and GII.4-2006a (Figure 5B).

### 3.3. RV VP6 Adjuvant Effect on Bivalent NoV VLP Immune Responses with Equal Doses of Antigens Used

**Figure 6:**
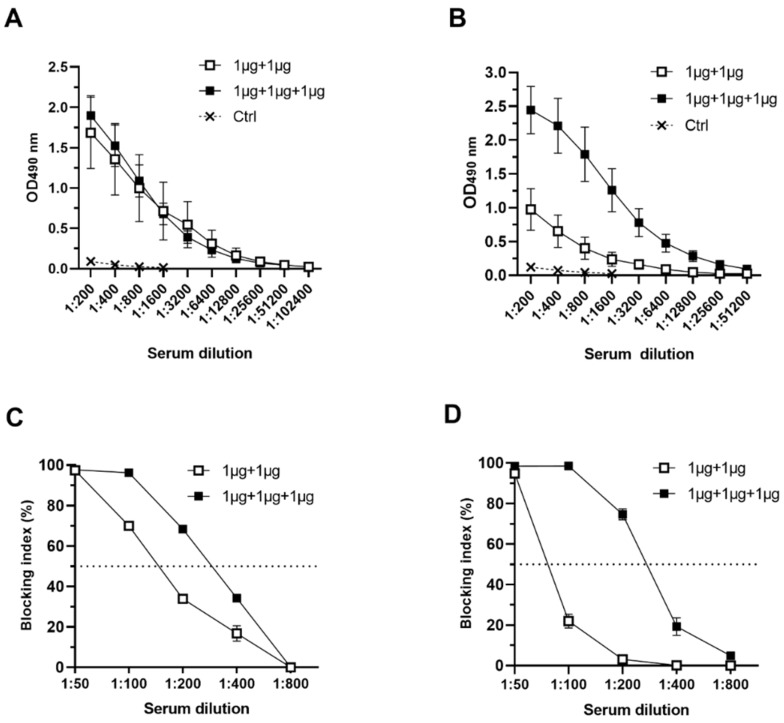
Serum norovirus (NoV)-specific antibody responses induced by equal doses of NoV VLPs and rotavirus (RV) VP6. Groups of mice immunized either with 1 µg of NoV GI.4 and GII.4-2006a VLPs or combined with 1 µg VP6 were individually tested for (**A**) GI.4- and (**B**) GII.4-2006a-specific serum IgG with two-fold dilutions starting at 1:200. Control (Ctrl) mice received carrier only. Pooled serum of both groups was tested for blocking of VLP binding using pig gastric mucin (PGM)–based assay. Shown are blocking titration curves of (**C**) GI.4 and (**D**) GII.4-2006a VLP binding. Horizontal dashed line represents 50% blocking.

To further investigate the VP6 adjuvant effect on bivalent NoV VLP response, the groups of mice were immunized using equal amount (1 µg) of each antigen. Mice receiving NoV GI.4 and GII.4-2006a VLP alone (Gr III, Table 1) generated GI.4-specific IgG response, which did not increase with VP6 co-administration (Gr IV, Table 1) (*p* > 0.05) (Figure 6A). GII.4-2006a-specific antibodies were also induced in mice immunized with 1 µg of NoV VLPs only (Figure 6B) (Gr III), but the level was quite low (GMT 1212). When 1 µg of VP6 was included to the vaccine formulation (Gr IV), GII.4-2006a-specific IgG response was significantly (*p* = 0.0317) improved (GMT 12800) (Figure 6B). Congruently with type-specific IgG titers, increase of cross-reactive GII-specific IgG antibodies against GII.4 SYD, GII.4 NO and GII.12 were observed when 1 µg of VP6 was co-administrated (Gr IV), but GI-cross-reactive IgG against GI.1 or GI.3 NoV VLPs were not improved (data not shown).

Group-wise pooled serum IgG of both experimental groups (III, IV) and the control group was analyzed for blocking activity of homologous GI.4 (Figure 6C) and GII.4-2006a (D) VLP binding to HBGAs present in PGM. There was slight increase in GI.4 VLP blocking activity in serum of mice immunized with VP6 and NoV VLPs (BT_50_ = 200), compared to mice immunized with NoV VLPs only (BT_50_ = 100) (Figure 6C). Blocking of GII.4-2006a VLP binding was low when pooled sera of mice receiving only NoV VLPs were tested in PGM HBGA blocking assay (BT_50_ = 50) (Figure 6D). The sera of mice immunized with VP6-containing vaccine formulation showed improved GII.4-2006a VLP blocking activity (BT_50_ = 200) (Figure 6D).

### 3.4. Plant-Based RV rVP6 Is Taken Up and Activates APC

**Figure 7:**
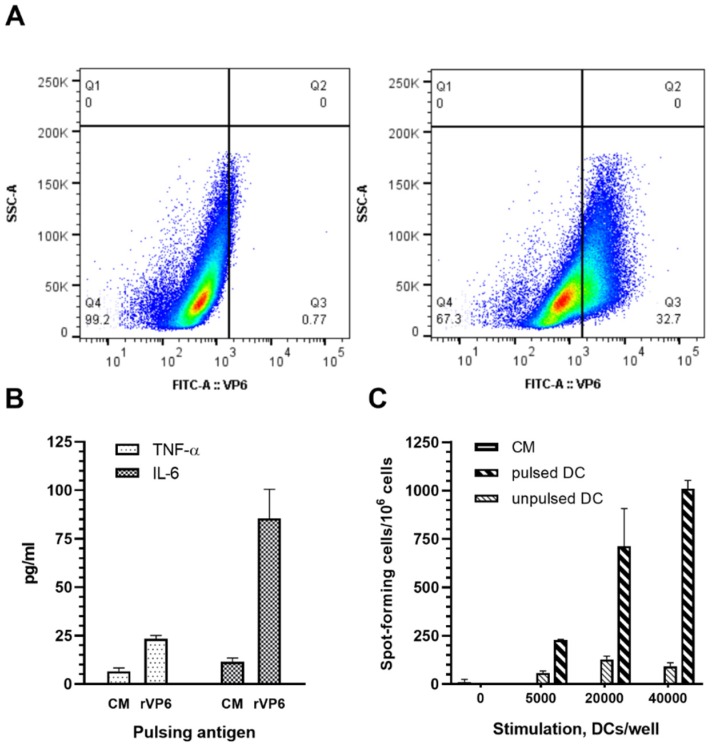
Rotavirus (RV) VP6 internalization, activation and presentation by bone-marrow-derived dendritic cells (BMDCs). Mouse BMDCs incubated for 20 h in the presence of (**A, left panel**) 100 µg/mL RV VP6 were intracellularly stained for RV VP6 and uptake of VP6 was analyzed by flow cytometry. BMDCs incubated in (**A, right panel**) culture media (CM) only served as negative controls. Uptake of VP6 is shown in the SSC-A versus VP6-FITC density dot plot on BMDCs gated on viable cells, a representative of repeated experiments. (**B**) TNF-α and IL-6 cytokines secretion by BMDC in response to VP6 was measured from CM collected at the end of the incubation period using ELISA. Shown are mean values (pg/mL) of the repeated analysis with standard errors of the mean. (**C**) VP6-pulsed BMDC were used at three different concentrations to stimulate VP6-specific mouse splenocytes in ELISPOT IFN-γ assay. Results are expressed as the mean spot forming cells (SFC) per 10^6^ splenocytes of the duplicate wells with standard errors of the mean.

Internalization of plant-based VP6 by BMDC used as APC was analyzed by intracellular staining following ~22 h incubation of the cells in the presence of 100 µg/mL RV VP6 protein. Approximately 33% of BMDCs had internalized VP6 during the incubation period (Figure 7A, left panel). Proinflammatory cytokine secretion by APC was measured in culture media collected at the end of the incubation period. VP6 stimulated BMDCs to produce IL-6, as well as TNF-α secretion (Figure 7B). Furthermore, APC processing and presentation of internalized VP6 to effector T cells were analyzed in ELISPOT IFN-γ assay using VP6-specific T cells as the responder cells. Strong IFN-γ response by T cells was observed correlating with increasing number of VP6-pulsed BMDC used for stimulation of the responder cells (Figure 7C).

## 4. Discussion

A dual role of BV expression system derived RV VP6 as a RV vaccine antigen and an adjuvant for monovalent NoV-specific immune responses in a NoV VLPs and RV VP6 combination vaccine has been previously published by our group [7,8,22,23]. The work was extended here to investigate if RV VP6 produced in another expression system improves the response of both, NoV GI and GII VLPs included into bivalent NoV VLP vaccine formulation. GI.4 and GII.4-2006a strains were selected based on their high incidence in human population, GII.4 variants have been predominant for the last two decades [36], while GI.4 is mainly encountered during foodborne outbreaks worldwide [37].

The suboptimal dose of 0.3 µg of both NoV VLPs was chosen based on our previous in vivo studies with monovalent baculovirus-derived NoV VLPs, indicating that the selected dose induces very poor NoV-specific immune responses [22,23], enabling VP6 adjuvant effect to be unambiguously addressed. Indeed, IgG antibodies to NoV GI.4 and GII.4-2006a were not detected in mice receiving bivalent NoV VLPs, but co-administration with 10 µg RV VP6 protein readily boosted both NoV GI.4- and GII.4-2006a-specific IgG responses. These IgG antibodies were able to prevent both NoV VLPs binding to cellular attachment factors, HBGA polysaccharides, which is so far the best correlate of protection from NoV infection [3,38,39]. Unfortunately, the lack of efficient NoV in vitro cultivation and animal challenge model prevents from showing in vivo protection of animals with the candidate vaccine. Human NoV neutralization assay may become possible via recent development of NoV culture system with human intestinal enteroid cells in near future [10]. Furthermore, co-administration with RV VP6 generated cross-reactive NoV-specific IgG antibody responses to six different GI and GII NoV genotypes tested. One of the major obstacles in induction of protective immunity to NoV infection is the heterogeneity of NoVs and therefore broadly cross-reactive immune responses are essential [11,12,40]. Clearance of NoV infection is most likely dependent on generation of both humoral and cell-mediated immunity [41,42,43,44,45]. Equal levels of serum NoV GI.4 and GII.4-2006a-specific IgG1, a marker of Th2 response, and IgG2a, a marker of Th1 response, were detected in groups that received bivalent NoV VLPs with 10 µg VP6, indicating that VP6 has an adjuvant effect on both arms of NoV-specific immunity. By contrast, the most commonly used adjuvants based on aluminum salts in several licensed VLP-based viral vaccines, mainly promote Th2-biased response, but have little capacity to stimulate cell-mediated immune responses [46].

We showed here for the first time that the VP6 adjuvant effect on NoV—specific immunity was observed also using equal doses of each NoV VLPs and RV VP6 antigen. As VLPs are extremely immunogenic in vivo and our previously published study showed that as low dose as 3 µg induces high IgG titer in mice, the dose of 1 µg of each antigen was used here [22]. Interestingly, using equal dosage of NoV VLPs in a bivalent formulation, we detected inferior GII.4-specific responses compared to GI.4 responses, that was, however, enhanced by co-administration with 1 µg RV VP6. Leroux-Roels et al. [9] have shown that GI.1-specific immune response interferes with GII.4c-specific responses in a clinical trial with equal dosage of NoV GI.1 and GII.4 VLPs. The authors reported that this immunological interference was corrected by increasing the dosage of GII.4 VLPs compared to GI.1 (a 3:1 ratio) but not by adding MPL as an adjuvant, in contrast to our observation with RV VP6. We have not detected any immune interference in comprehensive preclinical studies with our trivalent NoV–RV combination vaccine candidate (NoV GI.3 VLPs + GII.4-1999 VLPs + RV VP6, respectively) [7,8], further supporting the balancing and stabilizing effect of VP6 in the vaccine formulation.

Our earlier results using intramuscular administration route demonstrated that the VP6 adjuvant effect on NoV-specific immune responses is local, depending on co-delivery and co-localization of antigens [22,23]. Accordingly, in the present study using the vaccine formulated as a mixture of NoV VLPs and RV VP6 antigens we were able to demonstrate the VP6 adjuvant effect on bivalent NoV vaccine using another parenteral route, ID. VP6 might function as a carrier for NoV VLPs, e.g., by forming aggregates, that could explain the requirement of co-administration and co-localization both spatially and temporarily [22,23]. VP6 may also have stabilizing/depot effect on NoV VLPs similar to aluminum hydroxide (Alum), currently used in the most advanced NoV VLP vaccine in phase IIb clinical trials [9].

We have previously investigated the possible mechanism of BV expression system produced VP6 protein adjuvant effect on NoV VLP-specific immunity using mouse RAW 264.7 macrophage and JAWS II DC immortalized cell lines [24]. In the present study we assessed in vitro mechanisms for plant-based VP6 antigen, using primary mouse BMDC as APC [35]. Even though cell lines, such as JAWS II DCs, are more homogenous cell population and thereby results may be easier to reproduce [47], they may not reflect the events occurring in vivo as reliably as primary cells [48]. The results indicated that VP6 is efficiently internalized and presented by APCs and induces secretion of proinflammatory cytokines IL-6 and TNF-α, thereby likely contributing to induction of immune responses against NoV VLP components. Proinflammatory cytokines recruit more APCs to the injection site, thereby increasing the uptake and processing of co-delivered NoV antigens [24]. Congruently with our previous studies using JAWS II DCs, IL-6 secretion was shown to be higher than TNF-α [24].

The present study performed with plant-derived NoV VLPs and RV VP6 confirms previously observed BV-derived VP6 adjuvant effect on immune responses to both genotypes of bivalent NoV VLPs vaccine formulation, ruling out possible residual BV adjuvant effect. Live BV has been shown to increase NoV VLP induced responses by 10-fold [49] and possess strong immunostimulatory effects [50,51]. Finally, the results in here demonstrate that plant-based NoV VLPs and RV VP6 can be used as a subunit combination vaccine against childhood NoV and RV gastroenteritis and support the role of VP6 protein as an adjuvant that may replace external adjuvants in a pediatric vaccine.

## Figures and Tables

**Table 1:** Experimental and control groups of immunized mice. Mice were immunized intradermally (ID) at day 0 and 21 with the indicated dose at a volume of 50 µL/injection and terminated at day 35.

Group	Dose GI.4 + GII.4	Dose VP6	Mice/Group
I	0.3 µg + 0.3 µg	-	5
II	0.3 µg + 0.3 µg	10 µg	5
III	1 µg + 1 µg	-	5
IV	1 µg + 1 µg	1 µg	5
V (Control)	- ^1^	-	5

^1^ phosphate buffered saline (PBS), carrier only.
